# Management of Myocardial Infarction and the Role of Cardiothoracic Surgery

**DOI:** 10.3390/jcm13185484

**Published:** 2024-09-15

**Authors:** Shannon Parness, Panagiotis Tasoudis, Chris B. Agala, Aurelie E. Merlo

**Affiliations:** 1 Division of Cardiothoracic Surgery, Department of Surgery, School of Medicine, University of North Carolina at Chapel Hill, Chapel Hill, NC 27599, USA; shannon_parness@med.unc.edu (S.P.); aurelie_merlo@med.unc.edu (A.E.M.); 2 Department of Surgery, School of Medicine, University of North Carolina at Chapel Hill, Chapel Hill, NC 27599, USA

**Keywords:** myocardial infarction, cardiac surgery, CABG, PCI, post-MI complications

## Abstract

Myocardial infarction (MI) is a leading cause of mortality globally and is predominantly attributed to coronary artery disease (CAD). MI is categorized as ST-elevation MI (STEMI) or non-ST-elevation MI (NSTEMI), each with distinct etiologies and treatment pathways. The goal in treatment for both is restoring blood flow back to the myocardium. STEMI, characterized by complete occlusion of a coronary artery, is managed urgently with reperfusion therapy, typically percutaneous coronary intervention (PCI). In contrast, NSTEMI involves a partial occlusion of a coronary artery and is treated with medical management, PCI, or coronary artery bypass grafting (CABG) depending on risk scores and clinical judgment. The Heart Team approach can assist in deciding which reperfusion technique would provide the greatest benefit to the patient and is especially useful in complicated cases. Despite advances in treatment, complications such as cardiogenic shock (CS) and ischemic heart failure (HF) remain significant. While percutaneous coronary intervention (PCI) is considered the primary treatment for MI, it is important to recognize the significance of cardiac surgery in treatment, especially when there is complex disease or MI-related complications. This comprehensive review analyzes the role of cardiac surgery in MI management, recognizing when it is useful, or not.

## 1. Introduction to Myocardial Infarction

### 1.1. Overview

Myocardial infarction (MI) attributable to coronary artery disease (CAD) constitutes a significant global cause of mortality [1]. In the United States, MI occurs approximately every 40 s, resulting in around 580,000 new cases and 210,000 recurrent episodes annually [2]. MI can be categorized into ST-elevation MI (STEMI) and non-ST-elevation MI (NSTEMI). STEMI is characterized by complete occlusion of a coronary artery, leading to severe tissue ischemia and necrosis [3]. In contrast, NSTEMI involves partial occlusion, causing less extensive tissue damage [3]. The frequency of STEMI ranges between 30 and 40% while NSTEMI ranges between 60 and 70% [4]. Complications following MI are a major concern, including congestive heart failure (CHF) [5]. Another acute complication is cardiogenic shock (CS), with incidence rates of 5–10% in STEMI and 2–4% in NSTEMI cases, both carrying approximately 50% mortality rates [6]. Current guidelines emphasize reperfusion therapy as the cornerstone of MI management, involving medical therapy, percutaneous coronary intervention (PCI), or coronary artery bypass grafting (CABG) [3,7]. While PCI and medical management are first line for many of these presentations, CABG and cardiac surgery play a role in treatment of MI and complications post-MI.

### 1.2. Diagnosis of Myocardial Infarction

The 2021 American College of Cardiology (ACC)/American Heart Association (AHA) guidelines [7] will be referenced throughout this paper with specific classes and levels of recommendations included in Table 1, Table 2, Table 3, Table 4, Table 5 and Table 6. Upon arrival at the emergency room, patients with chest pain will require an electrocardiogram (ECG) to be performed. If deemed an MI, the ECG will distinguish between STEMI and NSTEMI which is imperative for deciding the best treatment route to take. If the ECG returns normal or nondiagnostic, serial ECGs should be performed, and a high-sensitivity troponin level should be monitored as well [8]. In addition to troponin, other labs to obtain include a complete blood cell count, comprehensive metabolic panel, and a fasting lipid panel [3]. Especially with thrombolytics being used for medical management of MI, knowing the platelet count and if there is evidence of anemia is important. Also, measuring electrolytes, especially potassium and magnesium, is crucial as imbalances can lead to arrythmias [3]. Electrical instability can occur if the conduction system of the heart is not being perfused. Therefore, depending on which vessel is affected, consequences such as heart block and tachyarrhythmias can further complicate the condition [9]. Along with clinical decision making, there are multiple grading systems to determine the best treatment method in these situations [3].

### 1.3. Non-ST-Elevation Myocardial Infarction vs. ST-Elevation Myocardial Infarction

STEMI is a higher acuity presentation than NSTEMI, with total occlusion of a coronary artery rather than a partial occlusion [3]. However, symptoms of these two etiologies are similar, and a distinction between them cannot be made until an ECG has been completed. STEMI diagnosis is made once a new ST-elevation is identified on ECG. On the other hand, NSTEMI diagnosis is made when a new ST-segment depression or T wave inversion is identified on ECG. Some patients with NSTEMI have normal ECGs, which is why serial ECGs, physical examination, and cardiac enzyme levels should all be taken into account before excluding this diagnosis [3]. STEMI is associated with numerous complications; for instance, CS occurs in 5–10% of patients [6], and heart failure (HF) is reported in 4–28% of cases [10]. Additionally, a study by Elbadawi et al. revealed that in 2015, mechanical complications occurred in 0.30% of STEMI patients, with a mortality rate of 42.4%, a statistic that remained unchanged since 2003 [11]. The severity of these complications and the hemodynamic instability observed in STEMI patients underscore the higher acuity nature of this presentation.

## 2. Treatment of Non-ST-Elevation Myocardial Infarction

### 2.1. Overview

**Figure 1:**
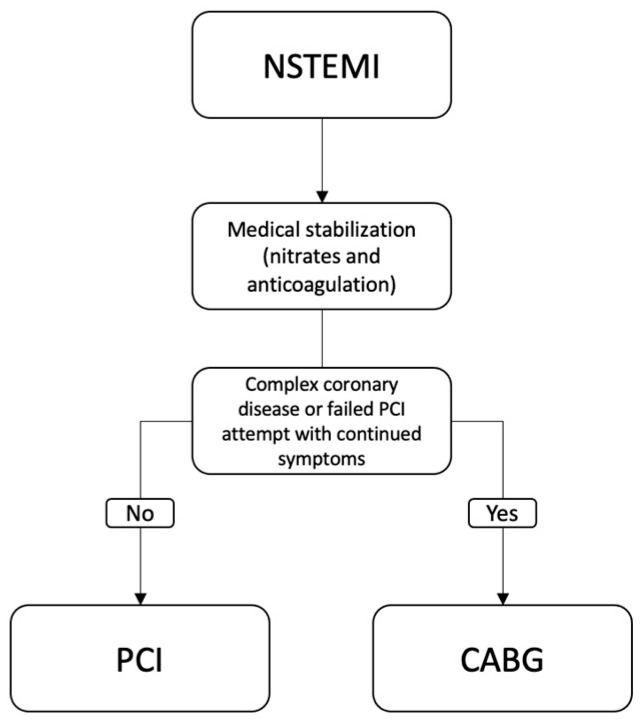
Adaptation of the 2021 ACC/AHA Indications for Revascularization in NSTEMI. Acronyms: ACC: American College of Cardiology, AHA: American Heart Association, NSTEMI: Non-ST-Elevation Myocardial Infarction, CABG: coronary artery bypass graft, PCI: percutaneous coronary intervention.

When presenting with an NSTEMI, initial medical management is important for stabilizing the patient. Fibrinolytics should not be used. Then, a strategy for revascularization should be determined; this can include PCI vs. CABG [3]. The 2021 ACC/AHA guidelines state that patients who are able to receive the procedure and are at an increased risk of future cardiac events should be revascularized using either PCI or CABG [7]. This can be measured using risk scores like Thrombolysis in Myocardial Infarction (TIMI), Global Registry of Acute Coronary Events (GRACE), or History, ECG, Age, Risk factors and Troponin (HEART). At a 4–6 month follow-up, treating with an invasive approach had a lower rate of death, MI, and refractory angina [7]. Also, patients who present with CS should be emergently revascularized; however, non-culprit vessel PCI should not be performed [7]. Intra-aortic balloon placement (IABP) may be beneficial in patients with CS who are planning to receive a CABG for revascularization [7,12]. If pregnant and presenting with an NSTEMI, PCI or CABG is reasonable if medical management is unsuccessful, and the patient’s life is at risk [7]. Figure 1 represents a simplified version of the 2021 ACA/AHA guidelines for revascularization in NSTEMI.

### 2.2. Heart Team

In most recommendations given by the 2021 guidelines in consideration of CABG vs. PCI, the Heart Team approach is mentioned as being a major part of the decision-making process to obtain the best results for each patient. Consisting of an interventional cardiologist, clinical cardiologist, and a cardiac surgeon, a Heart Team should be consulted when considering a reperfusion strategy [7]. This interdisciplinary group allows for shared decision making and finding the best treatment option for each patient. The team is able to consist of more physicians if necessary, like the patient’s primary care physician or any specialist they see. The best situations in which to use a Heart Team approach are those with complicated coronary disease, comorbidities present, and when social aspects have to be considered that may affect the outcomes of the procedure chosen. Overall, this team has been shown to improve clinical outcomes for patients, and it is recommended for hospitals to have a method in place to rapidly use this approach in urgent situations [7].

### 2.3. Factors Considered

Each patient is unique in their disease, so it is important to deliberate every aspect in order to choose the best treatment option. Factors listed that are taken into account by the Heart Team include coronary anatomy, comorbidities, procedural factors, and patient factors [7]. Within the coronary anatomy section, left main and multivessel disease are discussed, as well as the overall complexity of the coronary anatomy. The Synergy Between PCI with TAXUS and Cardiac Surgery (SYNTAX) Score is used to calculate the coronary complexity which will guide decision making for treatment options as well [7]. A few comorbidities considered include diabetes, systolic dysfunction, end-stage renal disease, and aortic aneurysm, among others. Procedural factors investigate the outcomes of the local and regional areas and the overall risk of procedure. Finally, patient factors include hemodynamic stability, patient preferences, ability to adhere to medical management, social support, and patient understanding [7]. Each factor is carefully examined and discussed within the Heart Team model to ensure the best treatment option is chosen.

### 2.4. Risk Scores

The HEART, TIMI, and GRACE scores assist in risk stratification for patients presenting with chest pain. These grading systems integrate additional patient risk factors, including age, ECG findings, and cardiac enzyme levels in order to develop the next steps in patient care [13]. For each assessment, the higher the score the greater the risk. A score is considered low risk when it equals zero in the TIMI score, when it is less than or equal to 72 in the GRACE score, and when it is less than or equal to three in the HEART score [13]. Each of these scores can be calculated upon patient presentation as the elements are based on basic patient characteristics and history. The HEART score is the only one that adds a consideration of physician opinion in its calculation [13]. The use of these systems is very important for clinical triage of patients. It considers patient risk factors, as well as provider opinion in the HEART score, to rapidly assist medical professionals with their decision making. A study conducted by Poldervaart et al. investigated the usefulness of these scores in identifying the risk of major adverse cardiac events (MACEs). They found that the HEART score performed the best in predicting a MACE compared to the TIMI and GRACE scores [13]. While risk scores provide valuable information, clinical judgements must be considered when deciding patient care. The integration of these expert opinions and risk scores allows for more successful care utilization of reperfusion techniques in treating patients with MI.

### 2.5. Percutaneous Coronary Intervention vs. Coronary Artery Bypass Grafting and Timing

The primary goal in NSTEMI treatment is restoring sufficient blood flow to the myocardium. In order to achieve reperfusion, there are many considerations when deciding between CABG and PCI. PCI is the most common reperfusion technique, especially in non-left main and non-complex disease, as it is less invasive, faster, and CABG has not been shown to demonstrate long-term survival benefit in this subgroup of patients [7]. Any patients who are at an increased risk of future cardiac events, calculated by the previously described risk scores, should be urgently revascularized using PCI or CABG [7]. There are times when CABG is the preferred method for revascularization in these patients. When there is complex coronary disease, such as left main or multivessel disease, it is recommended to choose CABG over PCI due to improvements in long-term survival [7]. It is also recommended to revascularize with CABG when there has been a failed PCI and there are ongoing ischemic symptoms, hemodynamic instability, or extensive myocardium at risk [7]. As the effectiveness of PCI reperfusion techniques increase, CABG procedures in the setting of MI have decreased, especially in treatment of single vessel disease [14].

As NSTEMI is a lower-acuity situation, the timing of treatment depends on the patient presentation. The risk scores can help decide when performing reperfusion therapy is most effective. Higher risk scores along with hemodynamic instability, threatening anatomy, or continued ischemic symptoms are associated with a more immediate need for revascularization. In addition, the presence of CS makes treatment an emergent situation [7]. If a risk score is calculated and the patient is considered high-risk, then treatment within 24 h is recommended. Finally, patients who are stable with intermediate or low risk scores can obtain their procedure prior to hospital discharge [7].

### 2.6. Medical Management

Early recognition of ischemic symptoms is very important so medical management can begin quickly and efficiently. Administering nitrates is considered first-line therapy. Nitrates vasodilate arteries and reduce preload, therefore reducing cardiac stress [3]. However, nitrates should not be given to those with a history of hypotension, right ventricular ischemia, severe bradycardia or tachycardia, and those who take phosphodiesterase inhibitors [3]. Antiplatelet therapy is another first-line medical therapy for these patients. Aspirin is typically administered to patients presenting with ischemic symptoms and another antiplatelet, specifically a P2Y_12_ inhibitor, is added before PCI and continued post PCI to reduce the chances of restenosis [3]. P2Y_12_ inhibitors include clopidogrel, prasugrel, and ticagrelor. While the standard timeline recommendation for dual antiplatelet therapy (DAPT) post-PCI is one year, if there is concern for major bleeding events, studies have shown that stopping aspirin between months one and three and continuing the P2Y_12_ inhibitor for the remaining years’ treatment time is reasonable [7].

## 3. Treatment of ST-Elevation Myocardial Infarction

### 3.1. Overview

**Figure 2:**
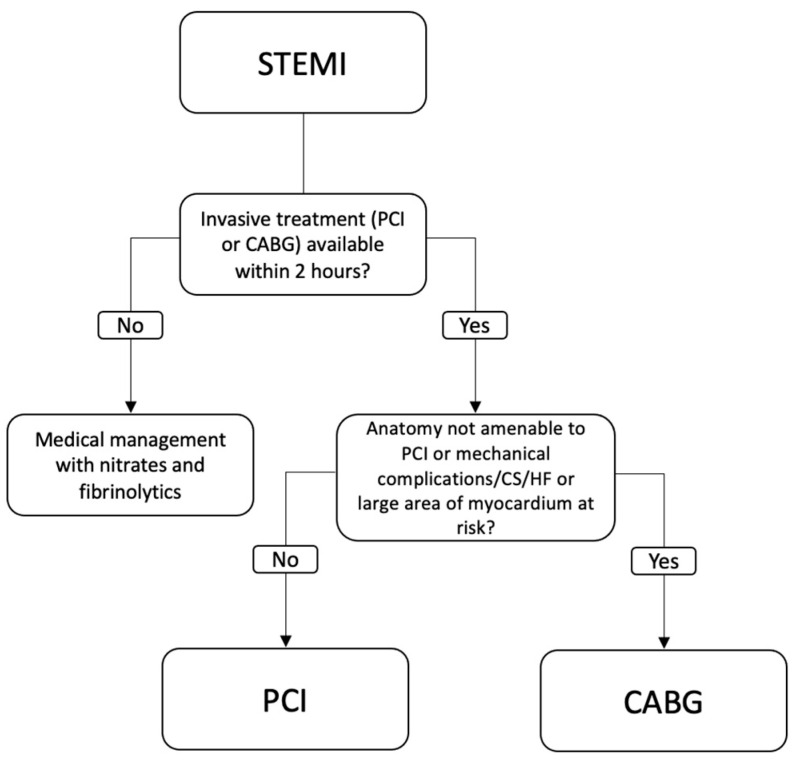
Adaptation of the 2021 ACC/AHA Indications for Revascularization in STEMI. Acronyms: ACC: American College of Cardiology, AHA: American Heart Association, STEMI: ST-Elevation Myocardial Infarction, CABG: coronary artery bypass graft, PCI: percutaneous coronary intervention, CS: cardiogenic shock, HF: heart failure.

Reperfusion therapy is the most important treatment for patients presenting with a STEMI. Options for reperfusion include medical management, PCI, and CABG. PCI is the preferred method for reperfusion and studies show that PCI has superior outcomes to fibrinolytic therapy [7]. Achieving blood flow back to cardiac tissue is crucial, so CABG is always a consideration when PCI or fibrinolytic therapy cannot obtain full reperfusion. CABG is indicated if the anatomy is not amenable to PCI, if a large area of myocardial tissue is at risk, or if there is evidence of CS, severe heart failure, or any mechanical complications. Mechanical complications include ventral septal rupture, papillary muscle rupture leading to valve insufficiency, free wall rupture, and ventricular aneurysm [3,7,15]. While the use of rapid reperfusion therapy has decreased the incidence of mechanical complications, mortality remained steady between the years of 1988 and 2008 (87.1% and 82.4%, respectively) [16]. Figure 2 represents a simplified version of the 2021 ACA/AHA guidelines for revascularization in STEMI.

### 3.2. When Percutaneous Coronary Intervention Is Recommended

When a diagnosis of STEMI is made, quick action needs to occur. Guidelines state that PCI should be performed if ischemic symptoms presented within the previous 12 h [7] and Bhatt et al. found that PCI within 120 min reduced mortality from 9% to 7% [15]. PCI is superior to treatment with medical management, and it has been shown to reduce major complications like death, stroke, MI, and bleeding [7]. If a patient presents to a hospital that is unable to perform a PCI, the recommendation is to transfer the patient to one that can perform it as long as the transfer time is practical and ischemic symptoms have been present for less than 2 h [7]. Also, if multivessel disease is present, after successful PCI of the culprit vessel and hemodynamic stability of the patient, PCI of non-culprit vessels with significant stenosis is recommended. However, patients presenting with CS should not receive non-culprit vessel PCI due to increased risk of death [7]. In pregnant patients, unless a spontaneous coronary artery dissection (SCAD) has occurred, PCI is preferred [7]. Rescue PCI is also performed for patients who have had failed reperfusion while being medically managed. It has been shown that after fibrinolytic therapy, performing a PCI reduces MACE and death [7]. Delayed PCI, classified as over 12 h after symptom onset, is not as well studied. The BRAVE-2 trial investigated the outcomes of treatment with PCI in asymptomatic STEMI patients that arrived 12–48 h after the onset of their symptoms. They found that PCI reduced the infarct size compared to treatment with medical therapy alone [17]. However, a totally occluded artery should not be treated with PCI if there is an extended amount of time between the onset of symptoms and arrival at the hospital. Therefore, depending on how long the delay to treatment is, PCI should be considered only when the artery is viable for treatment [7]. Nevertheless, if ischemic symptoms continue to be present, or there is evidence of severe HF or life-threatening arrythmia, then PCI can be performed irrespective of time to treatment [7].

### 3.3. When Medical Management Is Considered

Acute management with nitrates can assist in STEMI patients presenting with hypertension and heart failure [3]. However, fibrinolytic therapy is only recommended when PCI cannot be achieved within 2 h [7]. This is due to 35% of patients not achieving full reperfusion from fibrinolytics alone and another 10% receiving ineffective reperfusion [7]. Therefore, while medical management is used as an adjunct therapy for STEMI patients, it is rarely used alone as a primary revascularization technique. Medical management post-PCI has the same recommendations as NSTEMI treatment. This includes DAPT for one year unless there is concern for a major bleeding event, in which case aspirin can be discontinued at one to three months with P2Y_12_ monotherapy for the remainder of the treatment time [7].

### 3.4. When Surgery Is Considered

Surgery for STEMI patients is difficult as many arrive hemodynamically unstable. However, as revascularization is crucial, if other techniques are not achieving this goal, then surgery is the best option. When symptoms have been present less than 12 h and PCI is not feasible due to the coronary anatomy or the extent of coronary disease, then CABG is recommended [7]. Also, in a situation where PCI failed to revascularize the tissue and there is a large area of myocardium at risk of ischemia, CABG is recommended to restore blood flow [7]. If a patient has complex multivessel non-culprit artery disease, after successful revascularization with PCI of the culprit vessel, elective CABG can be completed to reduce the risk of a MACE. However, emergency CABG should not be performed after a failed PCI attempt if there are no ischemic symptoms, a large amount of myocardium is at risk, or if there are no distal targets for CABG to be successful [7]. Surgery is considered less often than PCI due to the added risks, but whenever PCI cannot be performed or does not accomplish reperfusion, then CABG is always a consideration.

When mechanical complications due to STEMI arise, CABG is one of the few effective options available for treatment of these patients, even though the mortality rate is high. The use of a mechanical support device, like IABP, may assist in delaying further complications, but surgery is the best option [7,12,18]. If the patient is too hemodynamically unstable to receive a CABG when indicated, mechanical circulatory support devices, like ventricular assist devices (VADs), can be used [3]. CABG is seemingly underutilized in those who would benefit from it. One study found that two-thirds of patients that had left main or multivessel disease did not receive a CABG in New York hospitals. New York has a “report card” system and, compared to other states that do not have this system, they were less likely to perform a CABG in an acute MI situation [19]. Overall, it is important to recognize when CABG better fills the role for reperfusion in STEMI patients even though PCI is the more popular option.

## 4. Post Revascularization Shock

### 4.1. Overview

Even after treatment of an MI, there can still be unavoidable consequences. In the short term, CS can ensue, and is defined as failure of the heart to send blood to the tissues, likely a result of inadequate pump function [6]. While CS is seen in a small subset of the population who have MIs, around 5–10% of patients, it is a grave situation when it happens and is the leading cause of death in hospitalized patients with MI [20]. In cases of CS, it is recommended to emergently revascularize the patient with PCI or CABG, irrespective of the time to treatment [7]. As mentioned above, Zeymer et al. found that in both STEMIs and NSTEMIs, the mortality rate after CS is around 50% [6]. Interestingly, Kolte et al. found that there had been an increase in the incidence of CS from 2003 to 2010, but with the increasing use of early mechanical revascularization and IABPs, the in-hospital mortality rate decreased [20]. However, Shah et al. noted that there has been a decrease in CS-complicated MI upon hospital admission from 65.3% to 45.6% from 2005 to 2014 [21]. Overall, CS patients are hemodynamically unstable, so successful outcomes can be difficult to achieve, but with early recognition and action these patients are more likely to survive.

Telukuntla and Estep describe the different mechanical support options available for patients with CS. The options available are split into left ventricular (LV), right ventricular (RV), and biventricular support. Left-sided support devices include IABP, Impella, and TandemHeart. Right-sided devices include Impella RP, and TandemHeart RA-PA. Biventricular assist operates through extracorporeal membrane oxygenation (ECMO). Compared to the others, the TandemHeart device does not have enough data to support or contradict its use [22].

### 4.2. Intra-Aortic Balloon Pump

IABP allows for up to a 20% increase in cardiac output with increasing ability for coronary perfusion [22]. Major complications from this procedure include limb ischemia, infection, and balloon rupture, which occurs in 2.6% of cases [22]. The IABP-SHOCK II trial investigated the use of IABP vs. medical therapy prior to revascularization. It showed that IABP placement did not reduce 30-day mortality in patients with CS-complicated MI compared to medical management [23]. Overall, the use of IABP has decreased in patients with CS, going from 29.8% in 2005 to 17.7% in 2014 [21]. As other devices prove to be superior to IABP, it is possible it will begin to be used only as a bridge to other treatments.

### 4.3. Impella

Impella, a percutaneous left ventricular assist device (LVAD), assists with LV function by offloading the blood volume to allow for relaxation and decreased oxygen demand of the heart [22]. The ISAR-SHOCK trial showed that Impella implantation was superior to IABP in obtaining hemodynamic support in CS [24]. The PROTECT II study also found hemodynamic support to be superior with Impella use over IABP, but there was no difference in the incidence of MACEs at 30 days between the two devices [25]. The use of Impella increased between 2005 and 2014, rising from 0.1% to 2.6% [21].

Impella RP is targeted for RV support rather than left. The RECOVER RIGHT study investigated the use of Impella RP in patients with right heart failure, some of whom were post-MI CS. While the cohort size was small, only five patients studied overall were placed into the category of post-MI CS, the investigators stated that this device is overall safe and feasible for use in critically ill patients experiencing right heart failure [26].

### 4.4. Extracorporeal Membrane Oxygenation

Veno-arterial (VA) ECMO assists with hemodynamic stability by reducing preload and increasing afterload and peripheral organ perfusion [27]. Due to the increase in afterload, the use of ECMO is contraindicated in patients with aortic regurgitation, and LV distention is a possible complication [27]. The ECMO-CS clinical trial found that use of VA-ECMO in severe CS did not improve clinical outcomes when compared to patients who were initially treated with a conservative approach [28]. However, the use of ECMO increased from 2005 to 2014, from 0.3 to 1.8% [21]. Bleeding complications pose a major risk for this patient population as well. Some treatments for MI include DAPT adherence, and VA-ECMO requires additional anticoagulation to reduce clotting within the circuit [29]. Masi et al. found that 40% of post-MI patients on VA-ECMO had a severe bleeding episode, and one-third had a major coagulation disorder [29]. Furthermore, Tigano et al. investigated the effects of hyperoxemia within this patient population. They found that patients exposed to high levels of oxygen while on VA-ECMO had poor neurological outcomes [30]. These studies show the importance of patient monitoring while on VA-ECMO.

## 5. Surgical Treatment of Post-Myocardial Infarction Complications

### 5.1. Cardiogenic Shock

Due to the high prevalence of MI worldwide, many studies have investigated post-MI-related complications and the best treatments available when they occur. With CS, a more acute complication of MI, revascularization is the primary goal. The ACC/AHA guidelines state regardless of time to reperfusion, STEMI complicated by CS should receive emergency revascularization with CABG or PCI [7]. However, CABG is mainly reserved for patients with left main or multivessel disease [19]. It has been found that in cases of CS-complicated MI, people who receive early revascularization, PCI or CABG, have better survival, quality of life, and fewer symptoms of HF compared to those who receive initial medical management for treatment [31]. An analysis of the SHOCK trial studied the difference in clinical outcomes of CS-complicated MI depending on if CABG or PCI was used. It was found that even though the patients receiving CABG had more extensive disease and increased risk factors, PCI and CABG had similar survival rates at 30 days and 1 year [32]. It is important to note that the SHOCK trial was not randomized and the decision to perform CABG or PCI was made using internal judgment. This led to CABG being used primarily in higher-acuity patients while the outcomes were compared to the lesser-acuity patients receiving PCI [32,33]. Looking at the results in this light, the survival rates being similar point to the possibility of CABG continuing to be a fair treatment in these situations. Therefore, even though CABG is not the mainstay treatment in the setting of an acute MI, it is still important to recognize its role when it is needed. Overall, quick action to restore perfusion to the tissues is essential in patients with CS.

### 5.2. Ventricular Septal Rupture

Mechanical complications post-MI are a grave consequence. The use of reperfusion therapy has significantly decreased the incidence, with fibrinolytic therapy decreasing all types of mechanical complications to 0.2–0.3%, and PCI further reduces this rate. If the patient does not receive reperfusion therapy, these complications peak 3–7 days from symptom onset [16]. Ventricular septal rupture (VSR) occurs from pressure overload of the necrotic septum causing a simple or complex rupture and creating a left-to-right shunt. This leads to pump failure and hemodynamic instability as the body attempts to compensate for a decreasing cardiac output [16]. Surgical repair is needed as 90% of patients who do not receive treatment may die within a month. The use of mechanical support devices, like IABP, may be considered to assist in offloading the ventricle [16].

### 5.3. Papillary Wall Rupture

Papillary muscle rupture (PMR), specifically of the mitral valve, is another mechanical complication that can occur post-MI. The acuity of this complication depends on the degree of mitral regurgitation and LV dysfunction associated with the rupture [16]. Rupture of the posterior–medial papillary muscle is witnessed 3–12 times more than an antero-lateral rupture due to its singular blood supply by the posterior descending artery (PDA) [16,34]. PMR may be partial or complete, and Bouma et al. found that a complete rupture had a 42.1% in-hospital mortality while a partial rupture had a 13.8% mortality [34]. IABP placement may be necessary in these patients to assist with stabilization and achieving coronary perfusion [34], especially since CS or pulmonary edema can develop rapidly [16]. Valve surgery is necessary once this complication occurs, and while performing a CABG has been shown in some studies to improve survival, the overall benefit of performing these procedures at the same time remains unclear [34].

### 5.4. Free Wall Rupture

Free wall rupture (FWR), specifically LVFWR, is another life-threatening complication post-MI. As with the other mechanical complications, the incidence has decreased to 0.01–0.5% with reperfusion therapies, yet mortality rates remain between 39 and 92% [35]. There are three different types of FWR. Type I occurs in the acute setting, less than 24 h, with a sudden rupture of the myocardium. Type II is identified as the gradual deterioration of the infarct zone, eventually leading to a tear. Finally, Type III is associated with aneurysm formation of the LV [16]. Surgery is the only therapy to decompress the cardiac tamponade and attempt to salvage the wall of the ventricle [16,35].

### 5.5. Aneurysm

Left ventricular aneurysm is a post-MI complication that is seen in 30–35% of patients [36]. A major risk factor is not achieving blood flow back to the myocardium, and as the muscle wall weakens, it forms an aneurysm. These aneurysms are identified as either true or false. True aneurysms are more commonly located on the apical and anteroseptal walls while false aneurysms involve the posterior or diaphragmatic surfaces [36]. Treatment for LV aneurysm depends on the size and if the patient has symptoms. Asymptomatic patients can be medically treated with cardioprotective drugs, along with anticoagulation to prevent thrombus formation [36]. In symptomatic patients, Sui et al. concluded that CABG with ventricular resection had the best clinical outcomes compared to PCI and medical management [37].

### 5.6. Ischemic Heart Failure

Ischemia-related HF is a long-term complication post-MI. This is the most common etiology of HF in the world, and it is from systolic left ventricular (LV) dysfunction attributed to obstructive CAD [38]. As stated above, in the United States, MI is the main contributor to CAD-related CHF [5], and late LV dilation develops in around 20% of patients preceding CHF despite early reperfusion [39]. One study claimed that the HF detriment in post-MI patients was due to the LV size rather than the ejection fraction [40]. This same study further went on to show that surgical ventricular restoration (SVR) improved systolic function and there was a low rate of re-hospitalizations for CHF [40]. However, SVR is not easily assessed, and therefore other analyses have pointed out the difficulty in stating it is a clinically useful procedure [41]. Ischemia-related HF is a prominent burden for the post-MI population, and treatment options become more invasive depending on the degree of the disease. Treatments include CABG, LVAD placement, and HT, which are discussed in depth below.

## 6. Heart Failure: Low-Ejection-Fraction Coronary Artery Bypass Graft vs. Advanced Therapies

### 6.1. Coronary Artery Bypass Graft

Cardiac surgery plays a critical role in managing patients who progress to end-stage HF, offering therapeutic interventions for those with limited options at this advanced stage of the disease. CABG is one such intervention. The STICH trial evaluated the mode of death in patients with ischemic HF treated with CABG combined with medical therapy versus medical therapy alone. The findings indicated that sudden death and pump failure were the most frequent causes of cardiovascular mortality in this patient population. While CABG did not significantly reduce all-cause mortality, it did lead to a marked decrease in the incidence of these two modes of death at two years [42]. The trial also observed a reduction in fatal myocardial infarction (MI) within the CABG group; however, the investigators noted that the benefits of CABG were somewhat mitigated by an increase in post-procedure mortality [42]. Long-term outcomes for post-MI HF patients have also been explored, revealing that CABG offers survival benefits, particularly in patients with viable myocardium, even in cases of advanced HF, with long-term survival observed up to 15 years post-procedure [43]. However, CABG is not always beneficial to patients with HF. An analysis of the CASS study showed that patients who did not have angina symptoms before receiving a CABG did not have improvements in their HF symptoms [41]. Also, a decreased LV end diastolic pressure (LVEDP) was correlated with decreased survival, even if sufficient myocardial viability was present [43].

### 6.2. Left Ventricular Assist Device

In end-stage HF, characterized by persistent symptoms and frequent hospitalizations despite optimal medical therapy, treatment options become increasingly limited. These options include LVADs and heart transplantation (HT) [44]. Initially, LVADs were predominantly used as a bridge to transplantation; however, with technological advancements, LVADs are now employed as a primary therapy, offering a viable long-term solution for patients who may not qualify for HT. This development is particularly significant given the complexities and lengthy process of organ donation. Although advancements in medical management have improved HF outcomes, a substantial number of patients still progress to end-stage HF [45]. The REMATCH study investigated the use of an LVAD as a primary treatment for patients who did not qualify for HT, and they found a 48% relative reduction in the risk of death for patients who received an LVAD compared to those being medically managed. They also found a 27% absolute reduction in mortality at one year [46]. The largest concerns after placing an LVAD are infection and mechanical failure. The investigators noted that the relatively low survival rate of 23% found at a two-year follow-up could be attributed to these complications [46]. However, this study was carried out in 2001, and an article published in 2020 showed that patients on the HT waiting list with a VAD had a one-year survival of 70% and a five-year survival of 33.3% [47]. The MOMENTUM 3 trial published in 2022 found that the newest LVAD, which operates with centrifugal-flow vs. previous axial-flow models, had an overall survival rate of 58.4% at 5 years [48]. This shows that with time and medical advancements, survival rates are increasing and engineering advancements may offset the worry of mechanical failure, so treatment with VADs will aim to continue providing an option to those with end-stage HF. The complication of right heart failure (RHF) after LVAD placement is another consideration when discussing treatment options. RHF after LVAD placement is reported in 5–44% of patients, a wide range possibly due to the varying definitions of RHF [49].

### 6.3. Heart Transplant

HTs are the gold standard treatment option for a patient with advanced HF. The limitations presented with an HT are the lack of donors and the life-long immunosuppressants patients must take afterwards [44]. These make qualifying and successfully receiving an HT very difficult to achieve. The survival rate after receiving an HT was 50.2% at 10 years, 30.1% at 15 years, and 17.2% at 20 years [50]. In that study, there were risk factors, like increasing recipient and donor ages, that negatively affected the survival rates. A recipient with zero risk factors had a 10- and 20-year survival of 59.7% and 26.2%, respectively [50]. These long-term survival rates are very promising, especially for an aging population. Cardiac surgery remains a viable treatment option for patients who have limited options once end-stage HF is reached, and it has played a large role in keeping these patients alive. PCI is the gold standard in acute MI treatment, but cardiac surgery is keeping its dominant role in the chronic management of a patient population that is ever growing.

## 7. Future Directions

It is important to recognize the gaps in knowledge when it comes to the role of cardiac surgery in MI and post-MI treatment. It is well established and studied that PCI and medical management have good clinical outcomes when treating acute MIs, but more research needs to be conducted for CS-complicated MI and the outcomes of revascularizing with CABG vs. PCI. Randomized trials would not be feasible in this situation, but retrospective studies could be of use to further knowledge of long-term outcomes depending on the revascularization technique. Also, more long-term outcome and survival studies are needed, especially for post-MI-related HF treatments. The STITCH trial extension study is one example that will be examining long-term outcomes depending on treatment of ischemic HF [42].

As medical advancements are occurring at an exponential rate and patients are surviving much longer than they did just 20 years ago, it is necessary to continue studying the long-term outcomes of LVAD placement. Dual et al. described how LVADs can be improved and detailed the current studies investigating the future of LVAD treatment [51]. For example, a long-term goal of LVAD placement is to have a completely implantable LVAD device. This way, the largest adverse event of driveline infection is no longer a concern. This has not yet been achieved as the issue of heat dissipation and tissue damage has not been solved [51]. Current improvements to LVAD devices include ways to minimize driveline infections by using smaller, more pliable drivelines or manufacturing drivelines with biocompatible material that will reduce infection rates [51]. Other aims include reducing the need for anticoagulation by limiting patient blood contact with LVAD machine elements, reducing the size of batteries required, creating a way to have physiologic control of the pump, and generating more LVAD sizes for a better fit in smaller patients [51]. There was already a large improvement in survival rates with LVADs between 2001 and 2020 [46,47], so continued research and engineering will continue to improve survival and allow LVADs to become a mainstay treatment for HF patients. Aside from LVADs, continued studies for HT are needed as well. Especially with an aging population, studies should keep investigating risk scores for those receiving an organ donation to better guide physicians and patients. These patients are surviving for decades after their surgery, an option that was not there for them prior to treatment, so long-term benefits and consequences of HT are still being explored. Another treatment option being explored for post-MI patients is the use of stem cells for myocyte regeneration. For example, induced pluripotent stem cells (iPSCs) and cardiac progenitor cells (CPCs) are being investigated for their ability to regenerate the damaged tissue post-MI [52]. Even though these studies are in the early stages, some show promise for the future. There is currently a role for cardiac surgery in MI treatment, particularly for post-MI complications. Present-day treatments coupled with quickly arising medical advancements appear to demonstrate that cardiac surgery will continue to hold a prominent role in MI-related treatments far into the future.

## Figures and Tables

**Table 1:** Adaptation of the 2021 ACC/AHA Guidelines for Class of Recommendation and Level of Evidence.

Class of Recommendation (COR)	Level of Evidence (LOE)
Class 1 (Strong): Benefit >>> Risk	Level A: high quality of evidence from multiple randomized control trials
Class 2a (Moderate): Benefit >> Risk	Level B: moderate quality of evidence from multiple randomized control trials or nonrandomized studies
Class 2b (Weak): Benefit ≥ Risk	Level C: limited data studies containing limitations of design or execution OR consensus of expert opinion based on clinical experience
Class 3 with LOE A or B (No benefit, Moderate): Benefit = Risk	
Class 3 without LOE A or B (Harm, Strong): Risk > Benefit	

**Table 2:** Adaptation of the 2021 ACC/AHA Guidelines for Patients with Chest Pain.

Class of Recommendation	Level of Evidence	Recommendations
1	C	Anyone experiencing acute chest pain should receive an ECG within 10 min of arrival to an emergency room

Acronyms: ACC: American College of Cardiology, AHA: American Heart Association, ECG: electrocardiogram.

**Table 3:** Adaptation of the 2021 ACC/AHA Guidelines for Patients with NSTEMI.

Class of Recommendation	Level of Evidence	Recommendations
1	A	Any patients who are at an increased risk of future cardiac events and can receive intervention should be revascularized using PCI or CABG
1	B	NSTEMI complicated by CS should be emergently revascularized with PCI or CABG
1	C	Patients with NSTEMI and hemodynamic instability should emergently receive invasive revascularization
2a	B	When PCI fails to revascularize the myocardium and there are ongoing ischemic symptoms, hemodynamic instability, or extensive myocardium at risk, CABG should be performed
2a	B	Patients who are initially stabilized with a high-risk score can receive invasive revascularization within 24 h
2a	B	Patients who are initially stabilized with an intermediate or low-risk score can receive invasive revascularization prior to hospital discharge
2a	C	In pregnant patients, PCI or CABG is reasonable if medical management is unsuccessful, and the patient’s life is at risk
3	B	NSTEMI complicated by CS should not receive non-culprit vessel PCI

Acronyms: ACC: American College of Cardiology, AHA: American Heart Association, NSTEMI: Non-ST-Elevation Myocardial Infarction, CABG: coronary artery bypass graft, PCI: percutaneous coronary intervention, CS: cardiogenic shock.

**Table 4:** Adaptation of the 2021 ACC/AHA Guidelines for Patients with Complex Disease.

Class of Recommendation	Level of Evidence	Recommendations
1	B	Use a Heart Team for clinical decision making when discussing reperfusion techniques
1	B	It is recommended to choose CABG over PCI when left main coronary disease is present
2a	B	It is recommended to choose CABG over PCI when multivessel disease is present
2b	B	Calculating the SYNTAX Score in patients with complex coronary disease can assist in treatment option decision making

Acronyms: ACC: American College of Cardiology, AHA: American Heart Association, CABG: coronary artery bypass graft, PCI: percutaneous coronary intervention, SYNTAX: Synergy Between PCI with TAXUS and Cardiac Surgery.

**Table 5:** Adaptation of the 2021 ACC/AHA Guidelines for DAPT in Patients Post PCI.

Class of Recommendation	Level of Evidence	Recommendations
2a	A	Post PCI, if there is concern for a major bleeding event, DAPT can be reduced to 1–3 months and the transition to P2Y_12_ monotherapy for the remaining treatment time is reasonable

Acronyms: ACC: American College of Cardiology, AHA: American Heart Association, PCI: percutaneous coronary intervention, DAPT: dual antiplatelet therapy.

**Table 6:** Adaptation of the 2021 ACC/AHA Guidelines for Patients with STEMI.

Class of Recommendation	Level of Evidence	Recommendations
1	A	If ischemic symptoms have been present less than 12 h, PCI is recommended
1	A	For patients with multivessel disease, if hemodynamic stability is attained, after successful PCI of the culprit vessel, subsequent PCI of stenotic non-culprit vessels is recommended
1	B	In CS complicated STEMI, emergently revascularize with PCI or CABG, irrespective of time to treatment
1	B	Patients with mechanical complications should receive CABG at time of surgery to improve survival
1	C	Patients with evidence of failed fibrinolytic therapy should receive rescue PCI of the culprit vessel
2a	B	Patients who are stable and presenting with STEMI 12–24 h after symptom onset can receive PCI
2a	C	In patients with complex multivessel non-culprit artery disease, after primary PCI of the culprit vessel, elective CABG can be carried out to reduce MACEs
2a	C	In pregnant patients, PCI is preferred unless SCAD has occurred
2a	C	PCI can be considered in patients with ongoing ischemia, severe HF, or life-threatening arrythmia, irrespective of time to treatment
3	B	CS complicated STEMI should not receive PCI of non-culprit vessels
3	B	Asymptomatic stable patients should not receive PCI if presenting >24 h after symptom onset and no evidence of severe ischemia
3	C	Emergency CABG should not be performed after a failed PCI if there is no ischemia or large portion of myocardium at risk or if there are no distal targets

Acronyms: ACC: American College of Cardiology, AHA: American Heart Association, STEMI: ST-Elevation Myocardial Infarction, CABG: coronary artery bypass graft, PCI: percutaneous coronary intervention, CS: cardiogenic shock, SCAD: spontaneous coronary artery dissection, MACEs: major adverse cardiac events, HF: heart failure.

## Data Availability

The original contributions presented in the study are included in the article, further inquiries can be directed to the corresponding author.
